# Regional dissemination of NDM-1 producing *Enterobacter hormaechei* ST1740, with a subset of strains co-producing VIM-4 or IMP-13, France, 2019 to 2022

**DOI:** 10.2807/1560-7917.ES.2024.29.11.2300521

**Published:** 2024-03-14

**Authors:** Inès Rezzoug, Cécile Emeraud, Christophe Rodriguez, Jean-Michel Pawlotsky, Rémy A. Bonnin, Laurent Dortet

**Affiliations:** 1 Team "Resist" UMR1184 "Immunology of Viral, Auto-Immune, Hematological and Bacterial diseases (IMVA-HB)," INSERM, Université Paris-Saclay, CEA, LabEx LERMIT, Faculty of Medicine, Le Kremlin-Bicêtre, France; 2 Bacteriology-Hygiene Unit, Bicêtre Hospital, Assistance Publique-Hôpitaux de Paris, Le Kremlin-Bicêtre, France; 3 Associated French National Reference Center for Antibiotic Resistance: Carbapenemase-Producing Enterobacterales, Le Kremlin-Bicêtre, France; 4 Université Paris-Est-Créteil (UPEC), Créteil, France; 5 Department of Virology, Hôpitaux Universitaires Henri Mondor, Assistance Publique-Hôpitaux de Paris, Créteil, France; 6 INSERM U955, Team « Viruses, Hepatology, Cancer », Créteil, France

**Keywords:** carbapenemase, NDM, VIM, IMP, β-lactamase, Enterobacterales

## Abstract

**Background:**

From 2019 to 2022, the French National Reference Centre for Antibiotic Resistance (NRC) received a total of 25 isolates of *Enterobacter hormaechei* subsp. *hoffmannii* sequence type (ST)1740. All produced metallo-β-lactamase(s) and were from the Lyon area.

**Aim:**

To understand these strains’ spread and evolution, more extended microbiological and molecular analyses were conducted.

**Methods:**

Patients’ demographics and specimen type related to isolates were retrieved. All strains underwent short-read whole genome sequencing, and for 15, long-read sequencing to understand carbapenemase-gene acquisition. Clonal relationships were inferred from core-genome single nt polymorphisms (SNPs). Plasmids and the close genetic environment of each carbapenemase-encoding gene were analysed.

**Results:**

Patients (10 female/15 male) were on average 56.6 years old. Seven isolates were recovered from infections and 18 through screening. With ≤ 27 SNPs difference between each other’s genome sequences, the 25 strains represented a clone dissemination. All possessed a chromosome-encoded *bla*_NDM-1_ gene inside a composite transposon flanked by two IS*3000*. While spreading, the clone independently acquired a *bla*_VIM-4_-carrying plasmid of IncHI2 type (n = 12 isolates), or a *bla*_IMP-13_-carrying plasmid of IncP-1 type (n = 1 isolate). Of the 12 isolates co-producing NDM-1 and VIM-4, seven harboured the colistin resistance gene *mcr9.2*; the remaining five likely lost this gene through excision.

**Conclusion:**

This long-term outbreak was caused by a chromosome-encoded NDM-1-producing ST1740 *E. hormaechei* subsp. *hoffmannii* clone, which, during its dissemination, acquired plasmids encoding VIM-4 or IMP-13 metallo-β-lactamases. To our knowledge, IMP-13 has not prior been reported in Enterobacterales in France. Epidemiological and environmental investigations should be considered alongside microbiological and molecular ones.

**Key public health message**

**What did you want to address in this study?**

Metallo-β-lactamases (MBL) called NDM-1, VIM-4 and IMP-13 can make bacteria resistant to many antibiotics. MBL are encoded by *bla* genes on chromosomes or plasmids. Whereas several bacterial species can harbour NDM-1 and VIM-4, IMP-13 mainly occurs in the *Pseudomonas aeruginosa* species, found in the environment. We investigated a French regional outbreak due to an NDM-1-producing strain of the species *Enterobacter hormaechei*.

**What have we learnt from this study?**

An *E. hormaechei of* subspecies *hoffmannii* ST1740 clone, with a chromosome-encoded *bla*_NDM-1_ caused the outbreak between 2019 and 2022, which happened in hospitals of the Lyon area. When spreading, the clone independently acquired *bla*_VIM-4_ or *bla*_IMP-13_ on respective IncHI2- and IncP-1-type plasmids. Enteric *E. hormaechei* can occur in the environment, so plasmid transfer between *P. aeruginosa* and the clone may have happened.

**What are the implications of your findings for public health?**

*E. hormaechei* subsp. *hoffmannii* belongs to the order of the Enterobacterales. Prior to this outbreak, IMP-13-producing Enterobacterales had not been observed in France. The outbreak source was probably in the environment, and environmental reservoirs should be considered in long-term outbreaks. Our findings highlight the need for epidemiological and environmental investigations to complement molecular and microbiological findings.

## Introduction

Carbapenems are often considered as last-resort antibiotics for the treatment of infections caused by Gram-negative bacilli. Since 2010, however, the prevalence of carbapenem-resistant Enterobacterales (CPE) has dramatically increased, mostly due to the global dissemination of carbapenemase producers [1].

Genes encoding carbapenemases are mainly carried on plasmids. Carbapenemases have been identified in three (A, B, D) of the four (A−D) classes of the Ambler classification. Carbapenemases of Ambler class A are mainly represented by *Klebsiella pneumoniae* carbapenemase (KPC) enzymes, which occur worldwide (mostly in China, Greece, Italy, South America and the United States) [2]. The majority of the Ambler class B enzymes, also named metallo-β-lactamases (MBLs), are represented by New Delhi MBLs (NDM)-, Verona integron-encoded MBLs (VIM)- and imipenemases (IMP). VIM producers are highly prevalent in Italy and Greece, while IMP producers are mostly reported from China, Japan and Australia. NDM producers, which originated from India have now disseminated globally [2]. Finally, in the west of Europe (including France), the most prevalent carbapenemases in Enterobacterales are oxallicinase (OXA)-48-like enzymes that belong to the Ambler class D. 

Contrary to Ambler class A and D carbapenemases, that possess an active serine in their catalytic site, class B carbapenemases (MBLs) require the binding of two zinc ions inside this site to be active [3]. MBLs are resistant to all commercially available β-lactamase inhibitors such as avibactam, clavulanic acid, relebactam, sulbactam, tazobactam and vaborbactam and hydrolyse all β-lactams except aztreonam. Unfortunately, MBL-producing strains frequently also produce extended-spectrum β-lactamases (ESBL) or over-express cephalosporinase, leading in both cases to successful aztreonam hydrolysis and to highly difficult-to-treat strains.

In France, during the last decade, *Enterobacter cloacae complex* (ECC) was often reported as the third most prevalent species among CPE behind *Escherichia coli* and *K. pneumoniae* [4]. ECC have been described to be ubiquitous in the environment and commensal enteric bacteria in humans [5]. However, they are also reported as opportunistic pathogens responsible for hospital-acquired infections [6]. Currently the ECC includes 19 species and six subspecies. These species are difficult to differentiate, because the most common identification method used in clinical microbiology, matrix-assisted laser desorption/ionization time-of-flight (MALDI-TOF), cannot efficiently discriminate them. Despite this, *E. hormaechei* subsp. *hoffmannii* has been reported as the most prevalent species among clinical and environmental ECC isolates [7,8].

The spread of carbapenemases within Enterobacterales is a common phenomenon associated with outbreaks. An example is the dissemination of several species of VIM-4 producing ECC in the Lyon area, which has been reported since 2015 [8]. Carbapenemases are also found in other Gram-negative bacilli such as *Pseudomonas aeruginosa*. Accordingly, IMP-13 has been reported nearly exclusively in *P. aeruginosa* isolates that have spread worldwide [9].

In this study, we investigated the dissemination in the Lyon area of ST1740 *E. hormaechei* subsp. *hoffmannii* isolates producing NDM-1 that were received from 2019 to 2022 at the Kremlin-Bicêtre French National Reference Centre (F-NRC) for antimicrobial resistance in carbapenemase-producing Enterobacterales.

## Methods

### Testing for carbapenemase production 

All isolates referred to the F-NRC are assessed for carbapenemase production with the Carba NP and NG Carba5 assays (NG Biotech, Guipry, France) as previously described [10,11]. Antimicrobial susceptibility testing is performed by disc diffusion on Mueller−Hinton agar (Bio-Rad, Marne la Coquette, France) and minimum inhibitory concentration (MIC) is determined by broth microdilution (ThermoFisher, Sensititre, France). In the current study, antimicrobial susceptibility results were interpreted according to European Committee on Antimicrobial Susceptibility Testing (EUCAST) guidelines as updated in 2023 [12].

### Whole genome sequencing, species confirmation and typing

For all carbapenemase producers identified at the F-NRC, whole genome sequencing (WGS) is conducted using the NextSeq500 system (Illumina technology) according to the manufacturer’s instructions. De novo assembly is performed with CLC Genomic Workbench v12,0 (QIAGEN, Les Ulis, France).

After MALDI-TOF, WGS data are employed to confirm the bacterial species, using Centrifuge Taxonomic Classifier 1.0.3 (https://github.com/chienchi/kbase-centrifuge). 

Multilocus sequence typing (MLST) analysis is performed using MSLT 2.0 server (https://cge.cbs.dtu.dk/services/MLST/). 

### Selection of bacterial isolates for the study

From 2019 to 2022, a total of 3,208 ECC isolates were received by the F-NRC (821, 692, 784 and 911 in 2019, 2020, 2021 and 2022, respectively), including 415 NDM producers (63, 71, 117, 164 in 2019, 2020, 2021 and 2022, respectively). Among the latter, 25 *E. hormaechei* subsp. *hoffmannii* clinical isolates of ST1740, which produced NDM-1, were included in the study. All these NDM-1-producing isolates originated from the Lyon area, France. Patient data such as age, sex (collected as binary variable: male/female), and hospitalisation ward were available with the isolates as well as information regarding the clinical sample (e.g. specimen type and date of isolation).

### Single nucleotide polymorphisms and phylogenetic analyses

The isolates included in the study were investigated using single nt polymorphisms (SNP) analysis. For this, sequence reads were mapped to a reference genome (GCF_001729745_ehormaechei_hoffmannii) using SNIppy v4.6.0 (https://software.cqls.oregonstate.edu/updates/snippy-4.6.0/). Metadata and phylogenetic trees (inferred by neighbour-joining method) were visualised using iTOL v6.5.2 (https://itol.embl.de/).

### Identification of antimicrobial resistance genes and plasmids

ResFinder server v4.0 (https://cge.cbs.dtu.dk/services/ResFinder/) was used to identify acquired antimicrobial resistance genes and PlasmidFinder (https://cge.cbs.dtu.dk/services/PlasmidFinder/) to detect plasmid replicon types. 

### Long read sequencing

Of the 25 isolates included in the study, long read sequencing was performed on 15. The latter had been selected to cover each subcluster or individual branch of the tree determined by phylogenetic analysis. The sequencing was achieved with Oxford Nanopore MinION technology (Oxford Nanopore, Oxford, United Kingdom), as previously described [13], to decipher the genomic localisation (chromosome of plasmid) of carbapenemase-encoding genes*,* and to analyse the genetic environment surrounding these genes. 

### Plasmids characterisation

Plasmids were reconstructed by combining Illumina and MinION sequencing data using Unicycler (v0.5.0). The assembled sequences were annotated using the Rapid Annotations using Subsystems Technology (RAST) server (rast.nmpdr.org). Integrons were classified according to INTEGRALL (http://intergrall.bio.ua.pt/) [14] and ISFinder (https://isfinder.biotoul.fr/blast.php). Plasmids’ visualisation was realised using the Proksee software (https://proksee.ca/).

### Conjugation experiments

Conjugation experiments were performed as previously described using the azide resistant *E. coli* J53 as recipient strain [15]. Transconjugants were selected on ticarcillin (50 μg/mL) and azide (100 μg/mL) supplemented agar plates.

## Results

### Patients’ characteristics

Of the 25 strains of ST1740 *E. hormaechei subsp. hoffmanni* received at the French NRC between 2019 and 2022, 10 were isolated from female and 15 from male patients. The average age of the patients was 56.6 years, and the median age was 65 years. Among the total 25 ST1740 ECCs strains, 18 were recovered from screening samples. The remainder seven were isolated because they were responsible for infections, including urinary tract infections (n = 2), bacteraemia (n = 1) and respiratory tract infections (n = 3). Among the respiratory tract infections, strains were isolated from bronchoalveolar lavage (n = 1), bronchial aspirations (n = 2) and sputum (n = 1). Infected or colonised patients were localised in the same geographical area, Lyon area (France), but hospitalised in three different hospital/departments. In detail, 18/25 were hospitalised at Hospital 1 in the infection disease department (n = 1), haematology department (n = 1), intensive care unit (ICU) (n = 2), paediatric cardiac resuscitation unit (n = 3), pneumology unit (n = 3), geriatric unit (n = 1), cardiology unit (n = 1) and adult surgery unit (n = 1), respectively. No data were available about the clinical ward for the last five patients hospitalised at Hospital 1. Three clinical strains were recovered from patients hospitalised at Hospital 2 (orthopaedics (n = 1) and ear, nose, and throat (n = 2) departments). Two isolates were from patients hospitalised at Hospital 3 in the ICU and rehabilitation care unit, respectively. Finally, two clinical isolates were cultured by two French private laboratories of the Lyon area from samples of patients who were no longer hospitalised; information on the hospitals where these patients had been prior admitted was not available.

### Long-term regional outbreak of NDM-1 +/− VIM-4 or IMP-13-producing *Enterobacter hormaechei*

**Figure 1:**
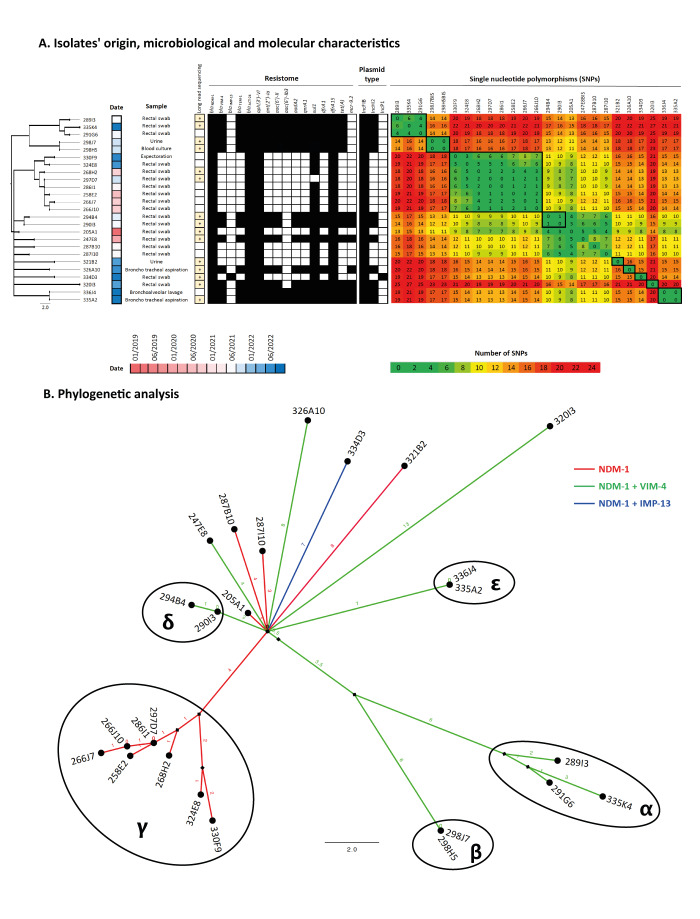
(A) Isolates’ origin, microbiological and molecular characteristics and (B) relationships between strains of NDM-1-producing *Enterobacter hormaechei* ST1740 analysed at the National Reference Centre for carbapenemase-producing Enterobacteriaceae, France, 2019−2022 (n = 25 isolates)

NDM: New Delhi metallo-β-lactamase; SNP: single nucleotide polymorphism; ST: sequence type.

All 25 strains were resistant to penicillin and their derivatives, and to 1^st^, 2^nd^, 3^rd^ and 4^th^ generation cephalosporins. β-lactamase inhibitors such as avibactam had no effect. MICs of imipenem and meropenem ranged from 1 to > 8 mg/L and from 1 to 16 mg/L, respectively. A total of 12 strains produced NDM-1 only and were isolated from patients hospitalised in the three different hospitals. This first group of strains included the oldest isolate which had been isolated in the first half of 2019 in Hospital 1 and strains in the group continued to be detected throughout the study period. Twelve other strains isolated from the three hospitals coproduced NDM-1 and VIM-4. The earliest isolate of this kind had been received by the F-NRC in the first half of 2020, with more such isolates until 2022. The last isolate coproduced NDM-1 and IMP-13 and had been isolated from Hospital 2 in the second half of 2022. The common resistome to all groups was composed of the carbapenemase-encoding *bla*_NDM-1_ gene, the intrinsic cephalosporinase encoding *bla*_ACT-5_ gene, and resistance determinants to aminoglycosides (*aph(3’)-VI*), fosfomycin (*fosA*) and trimethoprim (*dfrA15*) (Figure 1A). In addition to this common genome, the 12 VIM-4 producers also carried *bla*_TEM-1_, *ant(2”)-Ia*, *aac(6')-Il*, *aadA2*, *qnrA1*, *sul1*, *dfrA1* and *tet(A)*. Of note, among these 12 VIM-4 producers, seven had acquired the colistin resistance gene *mcr-9.2* including two that additionally acquired *aac(6')-Ib3*. Regarding the producer of NDM-1 and IMP-13, only the *bla*_IMP-13_ and *aac(6')-Ib3* genes, respectively encoding IMP-13 and conferring aminoglycoside resistance were added to the common resistome (Figure 1A).

A. Isolates’ origin, microbiological and molecular characteristics. A neighbour-joining phylogenetic tree is presented, with for each leaf, the date of strain isolation indicated in the first column (red for older strains to blue for more recent ones). The ‘+’ sign indicates MinION sequencing in the first column of the table. The presence of antimicrobial resistance determinants and plasmids are indicated in black for each strain. An SNP matrix is presented, with comparisons of strains in terms of differences in numbers of SNPs between them; those forming clusters in the phylogenetic tree are boxed.

B. Neighbour-joining phylogenetic tree of carbapenemase-producing ST1740 *E. hormaechei*. Clusters (α, β, γ, δ, ε) are circled. The distance between two strains is proportional to the number of SNPs. This number of SNPs is indicated on each line.

Since all NDM-1 +/− VIM-4 or IMP-13-producing *E. hormaechei* subsp. *hoffmannii* belonged the same sequence type (ST), we used a core genome SNP-based approach to create a phylogenetic tree (Figure 1). The maximum number of SNPs observed between two strains was of 27, suggesting that all these isolates were part of the same cluster that disseminated from January 2019 to September 2022. The comparison of SNPs numbers with spatio-temporal data of patients led us to identify five (α, β, δ, ε, γ) probable independent patient-to-patient transmission routes (Figure 1) corresponding to 17 isolates for which the number of SNPs were very low (0 to 8 SNPs). Phylogenetic investigations suggested a common source for all these isolates, which we were not able to definitively identify. However, the presence of *bla*_IMP-13_ (which is always reported in *P. aeruginosa*) suggests that *E. hormaechei* subsp. *hoffmannii* NDM-1 might have been present in the environment or contaminated it. Through an environmental route, this strain may also have come into contact with VIM-4-producing ECC that have been described to be highly prevalent in Lyon area since 2015 [8]. This scenario implies the transfer of *bla*_IMP-13_ or *bla*_VIM-4_-carrying plasmids in the ST1740 NDM-1-producing *E. hormaechei* subsp. *hoffmannii*. To confirm this hypothesis and characterise *bla*_IMP-13_ and *bla*_VIM-4_-carrying plasmids, long-read sequencing and conjugation experiments were performed.

### Characterisation of *bla*_NDM-1_ localisation and genetic context

**Figure 2:**
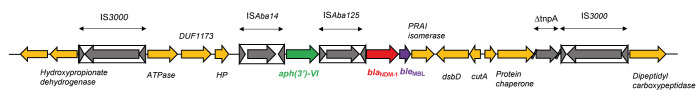
Linear map of the close genetic environment of the chromosome-encoded *bla*_NDM-1_ gene in an *Enterobacter hormaechei* ST1740 strain analysed at the National Reference Centre for carbapenemase-producing Enterobacteriaceae, France, 2019−2022

HP: hypothetical protein; IS: insertion sequence; NDM: New Delhi metallo-β-lactamase; PRAI: phosphoribosylanthranilate isomerase; ST: sequence type.

Whole-genome sequencing using a long-read technology was performed on 15 strains representative of all the sub-clusters identified this study (Figure 1A). The combination of long-read and short-read techniques allowed to reconstitute the chromosome of the ST1740 isolate, which was found to have a total length of ca 4,827,000 bp. The previously described common resistome of the 25 isolates was chromosome encoded, including *bla*_NDM-1_ which was carried on a composite transposon bracketed by two insertion sequences (IS)*3000* in all sequenced strains (Figure 2).

The carbapenemase-encoding gene is represented in red. The other antimicrobial resistance determinants are represented in green. Insertion sequences (IS) are represented in grey. The *ble*_MBL_ gene responsible to bleomycin resistance and systematically found downstream *bla*_NDM_ is represented in purple.

### Plasmids’ characterisation of ST1740 *Enterobacter hormaechei* subspecies *hoffmannii*

Replicases of several plasmids were identified in all the strains of this collection. IncFIB and col(pHAD28) replicases were observed in all strains, whereas IncHI2 was found only in the VIM-4-producing isolates (n = 12) and IncP-1 in the IMP-13-producing strain.

**Figure 3:**
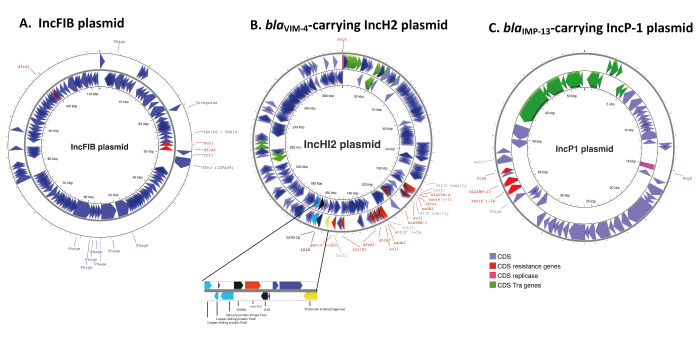
Circular representation of (A) an incFIB plasmid, (B) a *bla*_VIM-4_-carrying IncH2 plasmid and (C) a *bla*_IMP-13_-carrying IncP-1 plasmid found in *Enterobacter hormaechei* ST1740 strains analysed at the National Reference Centre for carbapenemase-producing Enterobacteriaceae, France, 2019−2022

Combination of long-read and short-read sequencing allowed to characterise these four plasmids. The col(pHAD28) plasmid was 2,495 bp and did not carry any resistance gene. The IncFIB plasmid was 112,496 bp and carried *dfrA1*, *dfrA15* and *sul1* genes conferring resistance to trimethoprim and sulfamethoxazole (Figure 3A).

CDS: coding sequence.

The representation was performed using Proksee software. Resistance genes are represented by red arrows. The *tra* genes implicated in plasmid self-conjugation are coloured in green. Other coding sequences are represented by purple arrows. The *mcr9.2* localisation into the *bla*_VIM-4_-carrying IncH2 plasmid is indicated under the panel B.

The *bla*_VIM-4_-carrying incHI2 plasmid was 298,456 bp in size. On this plasmid, the *bla*_VIM-4_ gene was located at the first position of a class 1 integron (In*416*) with *aacA7, dfrA1b* and *aadA1b* (Figure 3B). This plasmid also carried *bla*_TEM-1_, *ant(2”)-Ia*, *tet(A*), *aac(6’)-Ib3*, and *qnrA1*. Among the 12 incHI2 plasmid-carrying strains, seven additionally harboured the *mcr9–2* gene. This gene, responsible for colistin resistance, was integrated between two IS: IS*903B* and a truncated IS*1R* (Figure 3B). The five isolates that did not carry *mcr9.2* nevertheless kept IS*903B* and IS*1R*, suggesting an excision of *mcr9.2* from the IncHI2 plasmid in these strains. The incHI2 plasmids contained a transfer module (*tra* genes) involved in conjugation. Self-conjugation abilities were confirmed by transferring these plasmids in an *E. coli* J53 recipient strain.

Finally, IncP-1-type plasmid was of 52,000 bp in size and carried the *bla*_IMP-13_ gene. A similar (98.2% identity) IncP-1-type plasmid lacking the *bla*_IMP-13_ gene was previously described in *P. aeruginosa* (GenBank accession number: CP072561). This MBL encoding gene was bracketed by *aac(6’)-Ib3* (represented as *aac(6’)-Ib* on Figure 3C) conferring resistance to aminoglycosides, and the *fosX* gene conferring resistance to fosfomycin. Again, self-conjugation abilities conferred by the transfer module (*tra* genes) were confirmed experimentally.

## Discussion

This study provides molecular and microbiological characteristics of ST1740 *E. hormaechei* subsp. *hoffmannii* isolates that were involved in a long-term outbreak and that produced multiple carbapenemases. In all the 25 isolates of this outbreak, the *bla*_NDM-1_ gene was located on the chromosome. Although *bla*_NDM-1_ is most often reported as plasmid encoded in Enterobacterales, a chromosomal location of *bla*_NDM_ has already been described in *E. coli* [16], *Proteus mirabilis* [17], *K. pneumoniae* [18], and *P. vulgaris* [19], but never in ECC. Usually, mobile genetic elements carrying *bla*_NDM_ are derivatives from Tn*125* [20,21]. In the ST1740 outbreak clone, the *bla*_NDM-1_ gene was embedded into a composite transposon, Tn*3000* composed of two IS*3000* inserted inside a chromosome-encoded copy of this fossilised Tn*125* [22]. Remarkably, this *bla*_NDM-1_-carryingTn*3000* has been previously described on IncX3 and IncFIIκ plasmids in *E. coli* and in *E. hormaechei* subsp. *hoffmannii*, respectively [23].

Of note, VIM-4-producing ECC have been demonstrated to be endemic in the geographic area where the current outbreak took place since 2015 [8]. As observed in our ST1740 *E. hormaechei* subsp. *hoffmannii* clone, *bla*_VIM-4_ was most often carried on an IncHI2 plasmid inside a In*416* integron [8].

The *bla*_IMP-13_-carrying IncP-1 plasmid displayed 98.2% identity with pBYT1–2 plasmid (GenBank accession number: CP072561) recovered from *P. aeruginosa*. The difference between these two plasmids is in the acquisition of *bla*_IMP-13_, *aac(6’)-Ib* and *fos(X)* as integron cassette genes. First reported in Italy [24], *bla*_IMP-13_ has disseminated in *P. aeruginosa* worldwide [18] and is rarely described in Enterobacterales. To our knowledge, this is the first report of an IMP-13-producing *Enterobacter* spp. in France. 

Our genomic analysis combined with spatio-temporal data of patients highlighted a probable environmental source containing NDM-1-producing ST1740 *E. hormaechei* subsp. *hoffmannii.* The outbreak clone could have possibly spread via patients and/or environmental contamination and come into contact with VIM-4 producing ECC or IMP-13-producing *P. aeruginosa*. Since both *bla*_VIM-4_ and *bla*_IMP-13_-carrying plasmids have been demonstrated to be self-conjugative, we might hypothesise that *bla*_VIM-4_-carrying incHI2 plasmid and *bla*_IMP-13_-carrying IncP plasmid were transferred independently from endemic VIM-4-producing ECC and IMP-13-producing *P. aeruginosa* into the NDM-1-producing ST1740 *E. hormaechei* subsp. *hoffmannii* clone. Then, as shown in Figure 1B, all strains inside each sub-clusters are closely related suggesting that patient-to-patient transmission might have occurred independently with NDM-1, or NDM-1 together with VIM-4-producing ST1740 *E. hormaechei* subsp. *hoffmannii*. As previously described [25], environmental reservoirs such as toilets and tanks have to be considered in the spread of the clone in this long-term outbreak. Containment of such an outbreak requires not only strict infection control measures, but also an intensive cleaning process (descaling and bleaching) and sometimes material (toilets, tanks) replacement [25]. The F-NRC communicated its findings to the concerned hospitals.

A main limitation of this work is that the outbreak description is the result of a retrospective molecular analysis, and environmental investigations were not carried out to identify the source. Moreover, limited information was available on epidemiological links. In addition, this study is based on *E. hormaechei* subsp. *hoffmannii* strains received at the F-NRC, which may not constitute the complete set of isolates involved in the outbreak, since submission to F-NRC of bacterial isolates by clinical laboratories is done on a voluntary basis. Accordingly, even though no new *E. hormaechei* subsp. *hoffmannii* strains producing NDM-1 +/ − VIM-4 or IMP-13 have been received since September 2022, we cannot rule out that the environmental source has been removed and that the outbreak is not still silently active. 

### Conclusion

In this study, we described a long-term regional outbreak of chromosome encoded NDM-1-producing ST1740 *E. hormaechei* subsp. *hoffmannii* that subsequently acquired plasmid encoded VIM-4 or IMP-13 metallo-β-lactamases. This outbreak most probably involved environmental contamination where plasmid transfers occurred between this NDM-1-producing ST1740 *E. hormaechei* subsp. *hoffmannii*, endemic VIM-4-producing ECC and IMP-13-producing *P. aeruginosa.* To our knowledge, IMP-13, which mainly disseminates in *P. aeruginosa* has not been previously reported in Enterobacterales in France. Our results highlight the crucial need of environmental and epidemiological investigations to complement both microbiological analyses and whole genome comparisons, in order to more comprehensively elucidate outbreaks.
